# Activation of the cholinergic anti-inflammatory pathway by GTS-21 attenuates cisplatin-induced acute kidney injury in mice

**DOI:** 10.1371/journal.pone.0188797

**Published:** 2017-11-30

**Authors:** Prodyot K. Chatterjee, Michael M. Yeboah, Malvika H. Solanki, Gopal Kumar, Xiangying Xue, Valentin A. Pavlov, Yousef Al-Abed, Christine N. Metz

**Affiliations:** 1 Center for Biomedical Sciences, Feinstein Institute for Medical Research, Northwell Health, Manhasset, NY, United States of America; 2 Department of Medicine, Division of Nephrology, Medical College of Wisconsin, Milwaukee, WI, United States of America; 3 Elmezzi Graduate School of Molecular Medicine, Northwell Health, Manhasset, NY, United States of America; 4 Department of Pathology and Laboratory Medicine, Medical College of Wisconsin, Milwaukee, WI, United States of America; 5 Donald and Barbara Zucker School of Medicine at Hofstra/Northwell, Hempstead, NY, United States of America; 6 Center for Molecular Innovation, Feinstein Institute for Medical Research, Northwell Health, Manhasset, NY, United States of America; National Cancer Institute, UNITED STATES

## Abstract

Acute kidney injury (AKI) is the most common side effect of cisplatin, a widely used chemotherapy drug. Although AKI occurs in up to one third of cancer patients receiving cisplatin, effective renal protective strategies are lacking. Cisplatin targets renal proximal tubular epithelial cells leading to inflammation, reactive oxygen species, tubular cell injury, and eventually cell death. The cholinergic anti-inflammatory pathway is a vagus nerve-mediated reflex that suppresses inflammation via α7 nicotinic acetylcholine receptors (α7nAChRs). Our previous studies demonstrated the renoprotective and anti-inflammatory effects of cholinergic agonists, including GTS-21. Therefore, we examined the effect of GTS-21 on cisplatin-induced AKI. Male C57BL/6 mice received either saline or GTS-21 (4mg/kg, i.p.) twice daily for 4 days before cisplatin and treatment continued through euthanasia; 3 days post-cisplatin mice were euthanized and analyzed for markers of renal injury. GTS-21 significantly reduced cisplatin-induced renal dysfunction and injury (p<0.05). GTS-21 significantly attenuated renal *Ptgs2*/COX-2 mRNA and IL-6, IL-1β, and CXCL1 protein expression, as well as neutrophil infiltration after cisplatin. GTS-21 blunted cisplatin-induced renal ERK1/2 activation, as well as renal ATP depletion and apoptosis (p<0.05). GTS-21 suppressed the expression of CTR1, a cisplatin influx transporter and enhanced the expression of cisplatin efflux transporters MRP2, MRP4, and MRP6 (p<0.05). Using breast, colon, and lung cancer cell lines we showed that GTS-21 did not inhibit cisplatin’s tumor cell killing activity. GTS-21 protects against cisplatin-AKI by attenuating renal inflammation, ATP depletion and apoptosis, as well as by decreasing renal cisplatin influx and increasing efflux, without impairing cisplatin-mediated tumor cell killing. Our results support further exploring the cholinergic anti-inflammatory pathway for preventing cisplatin-induced AKI.

## Introduction

Cisplatin is a highly effective antineoplastic drug used for treating a myriad of solid tumors (e.g. testicular, ovarian, head and neck, breast, bladder, prostate, and others), with complete remission achieved in up to 90% of men diagnosed with stage III testicular cancer [1]. Despite its efficacy in treating numerous tumors, the use of cisplatin is limited by its adverse effects on normal tissues. Acute kidney injury (AKI), characterized by a sudden reduction in renal function together with the accumulation of metabolic waste and/or decreased urine output [2, 3], is the major dose limiting side-effect of cisplatin. AKI is observed in approximately 30% of patients receiving cisplatin, requiring dose reduction or discontinuation [4–6]. The pathophysiology of cisplatin-induced AKI is distinct from AKI induced by sepsis and ischemia reperfusion injury; it is characterized by cisplatin accumulation and DNA damage, as well as localized renal inflammation and oxidative stress, tubular epithelial cell apoptosis/necrosis, and eventually irreversible kidney damage [4, 7]. Except for supportive regimens (e.g. renal replacement therapy), there are no specific therapies available to consistently reduce or prevent cisplatin-induced AKI in these patients. Hence, identifying new agents for ameliorating local inflammation and oxidative stress associated with cisplatin-induced AKI may protect patients against cisplatin- nephrotoxicity in the future.

GTS-21, also known as 3-(2,4-dimethoxybenzylidene) anabaseine (or DMXB-A), is a selective α7 nicotinic acetylcholine receptor (α7nAChR) agonist. GTS-21 is a relatively safe drug candidate in humans and has been shown to significantly improve cognitive function and attention when compared to placebo [8]. Currently, GTS-21 is in clinical trials to evaluate its use for treating Alzheimer’s disease (NCT00414622), attention deficit disorder (NCT00419445), nicotine dependence (NCT02432066), and schizophrenia (NCT01400477). Although its precise mechanism of action in these conditions is not completely understood, GTS-21 is an activator of the efferent arm of the vagus nerve-based inflammatory reflex that is termed the cholinergic anti-inflammatory pathway. In this pathway efferent vagus nerve signaling with the subsequent release of acetylcholine controls cytokine production and inflammation via activation of α7nAChRs expressed on macrophages, endothelial cells and other immunocompetent cells [9–12]. In addition, the cholinergic anti-inflammatory pathway ameliorates oxidative stress in animal models and *ex vivo* [13–15]. Activation of the cholinergic anti-inflammatory pathway using α7nAChRs agonists attenuates inflammation in several organs, including the kidneys during ischemia reperfusion injury [13, 16, 17] and septic-AKI [18], as well as other inflammatory conditions including experimental models of colitis [19], atherosclerosis [20], and sepsis [21]. We have previously shown the constitutive expression of α7nAChRs in the kidneys and by renal tubular epithelial cells [16, 22]. Given that the activation of the cholinergic signaling is renoprotective by reducing inflammation and oxidative stress, we examined the efficacy of GTS-21 in alleviating cisplatin-induced AKI in mice. We report that administration of GTS-21 significantly attenuates renal inflammation, oxidative stress and ATP depletion, as well as renal tissue damage and apoptosis following cisplatin treatment. In addition, GTS-21 did not compromise the anti-tumor cell killing activity of cisplatin.

## Materials and methods

### Animals and cell lines

Male C57BL/6 mice (8–12 wks old) were purchased from Taconic Farms (Germantown, NY, USA). Animals were allowed to acclimate for at least one week prior to experimentation. All animals were housed under standard conditions (room temperature 22°C with a 12h light–dark cycle) with access to chow and water. All animal experiments were performed in accordance with the National Institutes of Health Guidelines and according to protocol #2009–002, which was approved by the Institutional Animal Care and Use Committee (IACUC) of the Feinstein Institute for Medical Research at Northwell Health. The LLC-PK_1_ porcine renal epithelial, MCF7 human differentiated breast cancer and CT26 mouse colon carcinoma cell lines were purchased from ATCC (Manassas, VA, USA). The H460 human large cell lung cancer cell line was provided by Dr. H Simpkins (The Feinstein Institute for Medical Research, Manhasset, NY, USA).

### Antibodies and other reagents

Phospho-ERK1/2 (p-ERK1/2, rabbit anti-mouse), total ERK1/2 (rabbit anti-mouse), MRP4 (rabbit anti-mouse) and GAPDH (rabbit anti-mouse) antibodies were purchased from Cell Signaling Technology (Danvers, MA, USA). MRP2 (rabbit anti-mouse) and MRP6 (rabbit anti-mouse) antibodies were purchased from Santa Cruz Biotechnologies (Dallas, TX, USA). GTS-21 was synthesized in the laboratory of Dr. Yousef Al-Abed (The Feinstein Institute for Medical Research, Manhasset, NY, USA). Cisplatin (cis-Dicholorodiammineplatinum(II)) was purchased from Acros Organics (Pittsburgh, PA, USA) and 2′, 7′-dichlorodihydrofluorescein diacetate (DCFH-DA) was purchased from Molecular Probes/Thermo Fisher Scientific (Waltham, MA, USA).

### Mouse model of cisplatin-induced AKI

C57BL/6 mice (males, 8–12 wks old, 20-25g, n = 8 mice per group) were acclimatized under normal environmental conditions and allowed free access to standard chow and tap water for at least 1 wk before experimentation. Mice were divided into 3 groups: (1) Control (CTRL); (2) cisplatin-treated (CIS) alone; and (3) GTS-21 plus cisplatin treated group (GTS+CIS). The GTS+CIS mice were injected with GTS-21 (4mg/kg, i.p. twice daily) for 4 days prior to cisplatin (20 mg/kg, i.p.) administration; GTS-21 treatments continued until euthanasia. Mice in the CTRL group received saline injections instead of GTS-21 and cisplatin. The mice were euthanized 72 hrs post- cisplatin (or saline) by CO_2_ asphyxiation/and exsanguination and blood was collected via cardiac puncture into heparinized needles/syringes; after centrifugation, plasma was collected and frozen at -80°C until analysis. Kidneys (outer medulla and cortex) were collected and either flash frozen in liquid N_2_ or fixed in 10% formalin.

### Determination of blood urea nitrogen (BUN) and creatinine levels

Plasma BUN and creatinine levels were determined using the respective Quantichrom kits (BioAssay Systems, Hayward, CA, USA), according to the manufacturer’s protocols.

### Real-time quantitative PCR (qPCR)

qPCR was performed using high quality RNA (OD 260/280 and 260/230 ≥2.0) isolated from frozen kidneys using the RNeasy Universal Plus Mini kit with elimination of genomic DNA (Qiagen, Valencia, CA, USA). qPCR reactions were performed in triplicate using the mouse *Ptgs2* (COX-2) primers (Roche Universal ProbeLibrary: F: 5′-GATGCTCTTCCGAGCTGTG-3′ and R: 5′-GGATTGGAACAGCAAGGATTT-3′), the Eurogentec One Step RT qPCR mastermix (AnaSpec, Fremont, CA, USA), 60ng RNA and the Roche 480 Light Cycler (Roche Diagnostics, Indianapolis, IN, USA). Mouse *Gapdh* was used as the housekeeping gene for normalizing transcript levels (F: 5’-GAGCCAAACGGGTCATCA-3’ and R: 5’-CATATTTCTCGTGGTTCACACC-3’). Changes in gene expression were calculated as relative fold-changes using the comparative Ct (ΔΔCt) method [23].

### Tissue chemokine and cytokine measurements

Approximately 100 mg kidney (cortex and outer medulla, frozen) per mouse was homogenized in 200μl lysis buffer (Tris buffered saline containing 0.25%Triton X-100 containing protease and phosphatase inhibitor cocktail, pH 7.4) in a Dounce homogenizer; supernatants were collected after centrifugation. Homogenates were assayed for cytokines and chemokines using a multiplex platform (Meso Scale Discovery, MSD) and the MSD Sector Imager 2400 plate reader (Meso Scale Diagnostics, Rockville, MD, USA). The electrochemiluminescent signals generated (raw data) were analyzed using the Discovery Workbench 3.0 software (MSD). The values were adjusted for protein concentration using the BioRad protein assay (Bio-Rad, Hercules, CA, USA).

### Tissue ATP and myeloperoxidase (MPO) levels

For assessment of ATP and MPO levels, kidneys (cortex and outer medulla) were homogenized in lysis buffer (as described above). ATP levels were measured using a colorimetric ATP assay kit (Biovision, San Francisco, CA, USA), according to manufacturer’s instructions. Tissue MPO levels were measured in the homogenate using an ELISA kit (Hycult Biotechnologies, Plymouth Meeting, PA, USA). Renal tissue ATP and MPO values were normalized to protein concentrations using the BioRad protein assay, as described above.

### Immunoblot analysis

Kidneys were homogenized in lysis buffer (as described above). Proteins (40–50μg/lane, determined using the BioRad protein assay) were separated by NuPAGE/SDS-PAGE electrophoresis (Invitrogen/Thermo Fisher Scientific, Waltham, MA, USA) and then transferred to Immobilon-FL PVDF transfer membranes (Millipore, Billerica, MA, USA). After blocking, the membranes were incubated with each primary antibody according to the manufacturer’s instructions. After washing, the blots were incubated with the appropriate near-infrared-fluorescently labeled secondary antibody (1:15,000, LI-COR Biosciences, Lincoln, NE, USA) for 45min and washed before revealing the bands using the Odyssey infrared imaging system (LI-COR Biosciences). Membranes were stripped using Re-blot Plus (Millipore) and re-probed with additional antibodies. Quantitation of the band densities was determined using Image J Software (NIH, Bethesda, MD, USA) and normalized using appropriate controls or housekeeping protein, GAPDH.

### Histological assessment of renal injury, neutrophil infiltration and renal cell apoptosis

Formalin-fixed kidneys were embedded in paraffin and sagittal sections (5μm) were prepared. To assess AKI-associated tubular injury, sections were stained with Periodic Acid-Schiff (PAS) (Sigma-Aldrich, St. Louis, MO, USA) and scored by a pathologist blinded to the experimental groups using a semi-quantitative scale to determine tubular injury (e.g. epithelial cell loss, necrosis, tubular epithelial simplification, intratubular debris and casts). Tubular injury scores (ranging between 0 and 4, using > 5 random fields/section, 6–8 mice per group) were based on the percentage of tubules affected (0: <10%; 1: 11–25%; 2: 26–50%; 3: 51–75%; 4: >75%), as previously described [24]. Leder staining was used for assessing neutrophilic infiltration using the Napthol AS-D Chloroacetate specific esterase kit (Sigma-Aldrich). The kidney sections were scored by counting the number of neutrophils per high power field (using > 5 random fields/section, 6–8 mice per group), as previously described [24]. Renal cell apoptosis in the cortex and outer medulla was measured by terminal deoxynucleotidyl transferase (TdT)-mediated dUTP nick-end labeling (TUNEL) using the ApoTag kit (Millipore). The sections were scored by counting the number of densely brown stained apoptotic cells per high power field (HPF), as previously described [24].

### Determination of kidney platinum (Pt) accumulation

Kidney platinum (Pt) levels were measured by inductively coupled plasma mass spectrometry (ICP-MS) at the University of North Carolina at Chapel Hill using an Agilent 7500cx (Agilent Technologies, Santa Clara, CA, USA), as previously described [24]. Pt concentrations were normalized to kidney weights.

### In vitro oxidative stress (DCFH-DA) and glutathione assays

DCFH-DA assay: LLC-PK_1_ renal epithelial cells were grown in M199 containing 10% fetal bovine serum (FBS), penicillin-streptomycin (PS), and glutamine (Q) to 90% confluency. After 24hrs, the media was replaced with minimum essential media (MEM) containing 5%FBS, Ca (0.2 g/L), Mg (97 mg/L, as MgSO_4_), non-essential amino acids and PSQ. On the next day, cells were treated with GTS-21 (1μM) or N-Acetyl Cysteine (NAC, 2mM). After 16 hrs, cells were harvested, resuspended (1x10^6^cells/ml) in either MEM (plus vehicle) or MEM containing cisplatin (50µg/mL) ± GTS-21 (1μM) or NAC (2mM) and incubated at 37°C/5% CO_2_ for 3.5 hrs. After the incubation period, cells were washed with HBSS and labeled with 2′,7′-Dichlorofluorescin diacetate (DCFH-DA, 20μM) for 20 min. Labeled cells were washed and analyzed for oxidative stress by the conversion of the non-fluorescent DCFH-DA into a fluorescent compound, 2′,7′-dichlorofluorescein (DCF) by ROS intermediates [25, 26], which was measured 30min later using the Victor3 fluorescence plate reader (Perkin Elmer) at Ex485/Em535 nm.

Glutathione assay: LLC-PK_1_ renal epithelial cells were grown as described above and cells were treated with vehicle or GTS-21 (1μM) for 1hr followed by the addition of cisplatin (0 or 30 μM). After 20hrs, cells were harvested and the amount of reduced glutathione (GSH) in the cell lysates was determined using a glutathione fluorometric assay kit (Biovision, San Francisco, CA, USA) and corrected for cellular protein using BCA Protein Assay Kit (Pierce/Thermo Fisher Scientific).

### Assessing the effect of GTS-21 on cisplatin-mediated killing of lung, breast, and colon tumor cell lines *in vitro*

H460 human large cell lung cancer cells were grown in RPMI 1640 media containing 10% FBS, PSQ in 96-well plates and allowed to reach ~60% confluence. The media was replaced with (MEM) containing 5%FBS, Ca (0.2 g/L), Mg (97 mg/L, as MgSO_4_), non-essential amino acids and PSQ. After 24 hrs, cells were pretreated with saline or GTS-21 (0, 1, 10μM). After 1 hr, vehicle or cisplatin (0–230μM) was added and cytotoxicity was measured using the MTT assay (OD_570/690_) 24 hrs later. The IC_50_ values for the tumor cell killing by cisplatin were calculated using non-linear regression to fit the data to the log (inhibitor-cisplatin dose) vs. response (variable slope-percent viable) curve using GraphPad Prism (GraphPad Prism Software, San Diego, CA, USA). The same procedures were followed for MCF7 human differentiated breast cancer and CT26 mouse colon carcinoma cell lines, except the MCF7 cell line was originally grown in DMEM containing 10% FBS, PSQ.

### Statistical analyses

Experiments were performed at least twice and data are expressed as mean±SD (or ±SEM), as indicated. One-way ANOVAs were used for multiple comparisons followed by Bonferroni post-hoc testing using GraphPad Prism (GraphPad Software, San Diego, CA). P values <0.05 were considered significant.

## Results

### Administration of GTS-21 protects against cisplatin-induced renal injury

**Fig 1:**
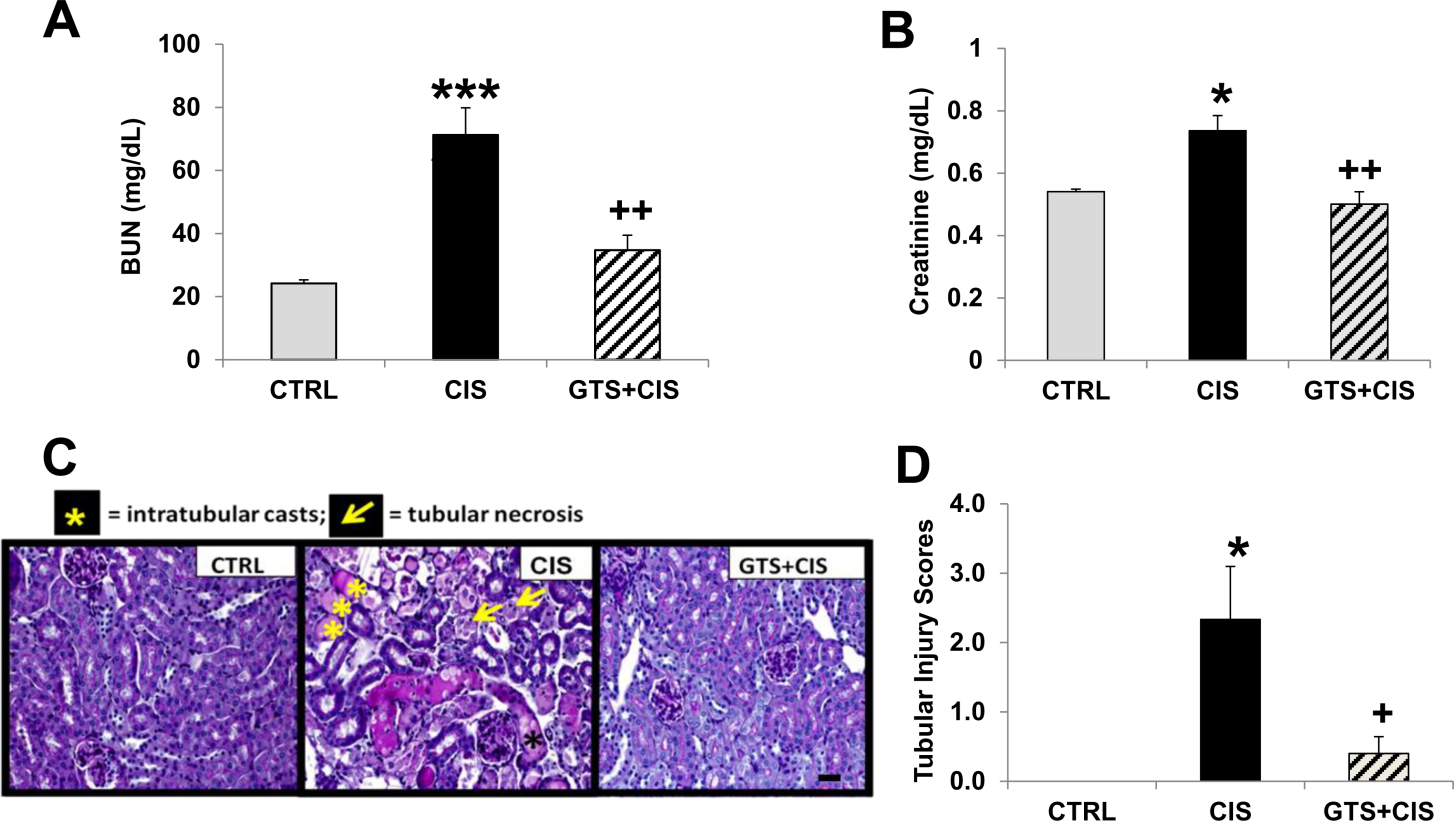
Cisplatin induces renal damage and GTS-21 exerts renoprotective effect. C57BL/6 mice (males, n = 8 mice per group) were injected daily for 4 days, prior to cisplatin (20 mg/kg, i.p.), with either saline (cisplatin only group, CIS) or GTS-21 (GTS-21 pretreatment group, GTS+CIS, 4mg/kg, i.p, twice) and until euthanasia 72hrs post-cisplatin. The control group (CTRL) received saline injections in place of GTS-21 or cisplatin. Kidney damage was assessed by measuring blood urea nitrogen (BUN, A) and plasma creatinine (B) levels. For tubular injury, fixed kidney tissues were stained with Periodic Acid Schiff reagent (PAS, C), scale bar, 50 μm; representative images for each group (x400) are shown and were scored for histological damage (0–4) (D). The scores were based on the percentage of tubules affected (0: <10%; 1: 10–25%; 2: 26–50%; 3: 51–75%; 4: >75%). Data are shown as mean ± SD. *** = p<0.001 vs. CTRL, * = p<0.05 vs. CTRL, ^++^ = p<0.01 vs. CIS, ^+^ = p<0.05 vs. CIS.

C57BL/6 mice treated with cisplatin alone exhibited elevated BUN (Fig 1A) and plasma creatinine levels (Fig 1B), as well as increased renal tubular injury (Fig 1C and 1D) when compared to control mice. Mice treated with GTS-21 prior to and after cisplatin (20 mg/kg, i.p.) showed significantly less cisplatin-mediated renal injury, as assessed by measuring blood urea nitrogen (BUN, Fig 1A) and plasma creatinine (Fig 1B) levels when compared to cisplatin-treated mice. Likewise, GTS-21 significantly blocked cisplatin-induced tubular injury, as determined by histological assessment of PAS-stained kidney tissues (Fig 1C and 1D).

### GTS-21 dampens cisplatin-induced kidney inflammation

**Fig 2:**
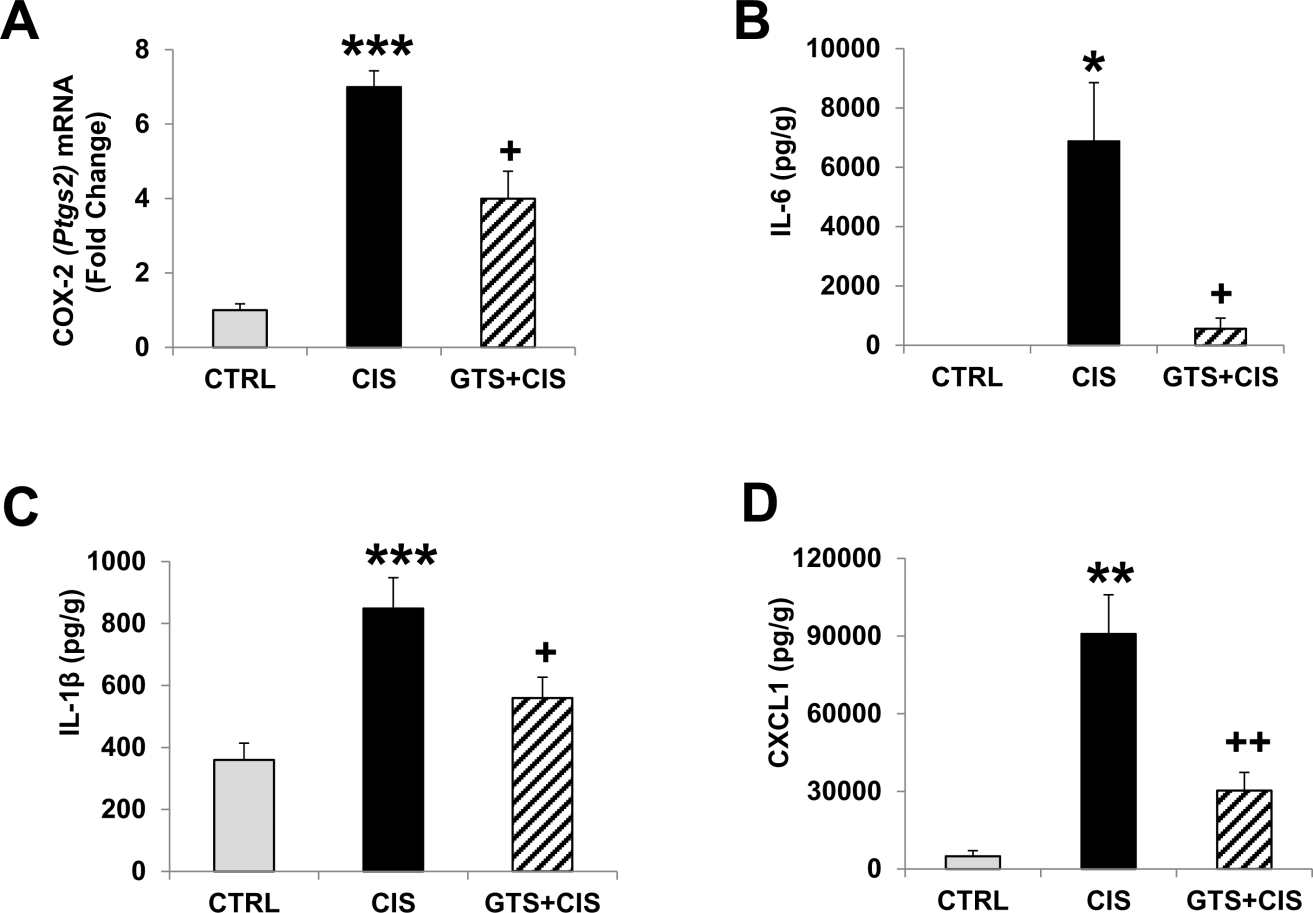
GTS-21 attenuates cisplatin-induced renal inflammation. Mice (CTRL, CIS and GTS+CIS groups, n = 8 per group) were treated and euthanized as described in the methods section and in Fig 1. COX-2 (*Ptgs2*) mRNA expression in the renal cortical tissues was measured by qPCR (A). Data are shown as mean (±SD) fold-change (vs. *Gapdh* housekeeping gene). Kidney homogenates were also analyzed for levels of inflammatory marker proteins (IL-6, B; IL-1β, C; and CXCL1, D) by Meso Scale Discovery (MSD) platform. Data are shown as mean (pg) per g of protein (±SD). *** = p<0.001 vs. CTRL, ** = p<0.01 vs. CTRL, * = p<0.05 vs. CTRL, ^++^ = p<0.01 vs. CIS, ^+^ = p<0.05 vs. CIS.

Renal injury following cisplatin administration is associated with the production of local inflammatory markers [4, 7]. Cisplatin dramatically increased renal *Ptgs2* (COX-2) mRNA expression (Fig 2A) and GTS-21 treatment significantly downregulated *Ptgs2* mRNA expression induced in the kidneys following cisplatin. Similarly, cisplatin administration led to the production of additional inflammatory proteins in the kidneys, including cytokines (IL-6 and IL-1β) and the chemokine CXCL1 (Fig 2B and 2D) over that observed in control kidneys. GTS-21 treatment significantly attenuated cisplatin-induced production of these inflammatory mediators in the kidneys (Fig 2B and 2D). TNF-α levels were not detected in any kidney tissues, whereas renal IL-10 levels were detected but the IL-10 concentrations were not significantly different among the three groups (control: 48±82pg/g; cisplatin alone: 26±42pg/g; cisplatin+GTS-21: 38±84pg/g).

### Cisplatin-induced neutrophil infiltration into the kidneys is blunted by GTS-21

**Fig 3:**
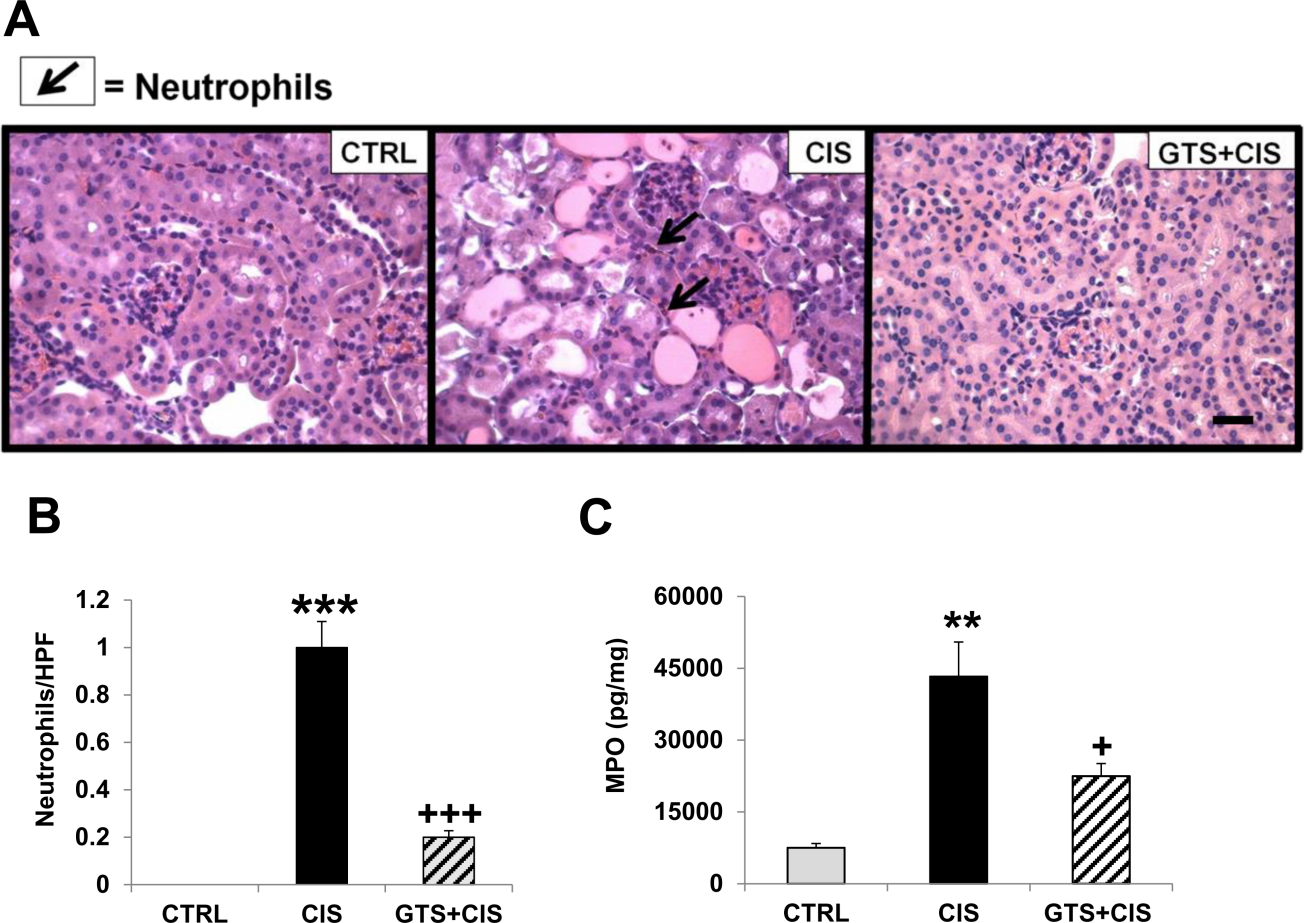
Cisplatin-induced renal neutrophil infiltration is blunted by GTS-21 pretreatment. Mice (CTRL, CIS and GTS+CIS groups, n = 8 per group) were treated and euthanized as described in the methods section and in Fig 1. Fixed kidney tissues were evaluated for neutrophils by Leder staining. Representative images for each group are shown in (A) at 400x magnification (scale bar, 50 μm) and the mean number of neutrophils per high power field, HPF (±SEM) is shown in (B). Frozen renal tissues were analyzed for myeloperoxidase (MPO) (C). Data are shown as mean MPO levels (pg) per mg protein (±SD). *** = p<0.001 vs. CTRL, ** = p<0.01 vs. CTRL, ^+++^ = p<0.001 vs. CIS, ^+^ = p<0.05 vs. CIS.

Consistent with the increased production of CXCL1, a neutrophil chemotactic factor, in the kidneys following cisplatin (Fig 2D), we observed enhanced renal neutrophil infiltration and MPO levels following cisplatin treatment when compared to the control kidneys (Fig 3A, 3B and 3C). Both renal neutrophil accumulation and MPO levels were significantly attenuated when mice received GTS-21 prior to and after cisplatin (Fig 3A, 3B and 3C).

### GTS-21 administration suppresses cisplatin-induced renal ERK activation

**Fig 4:**
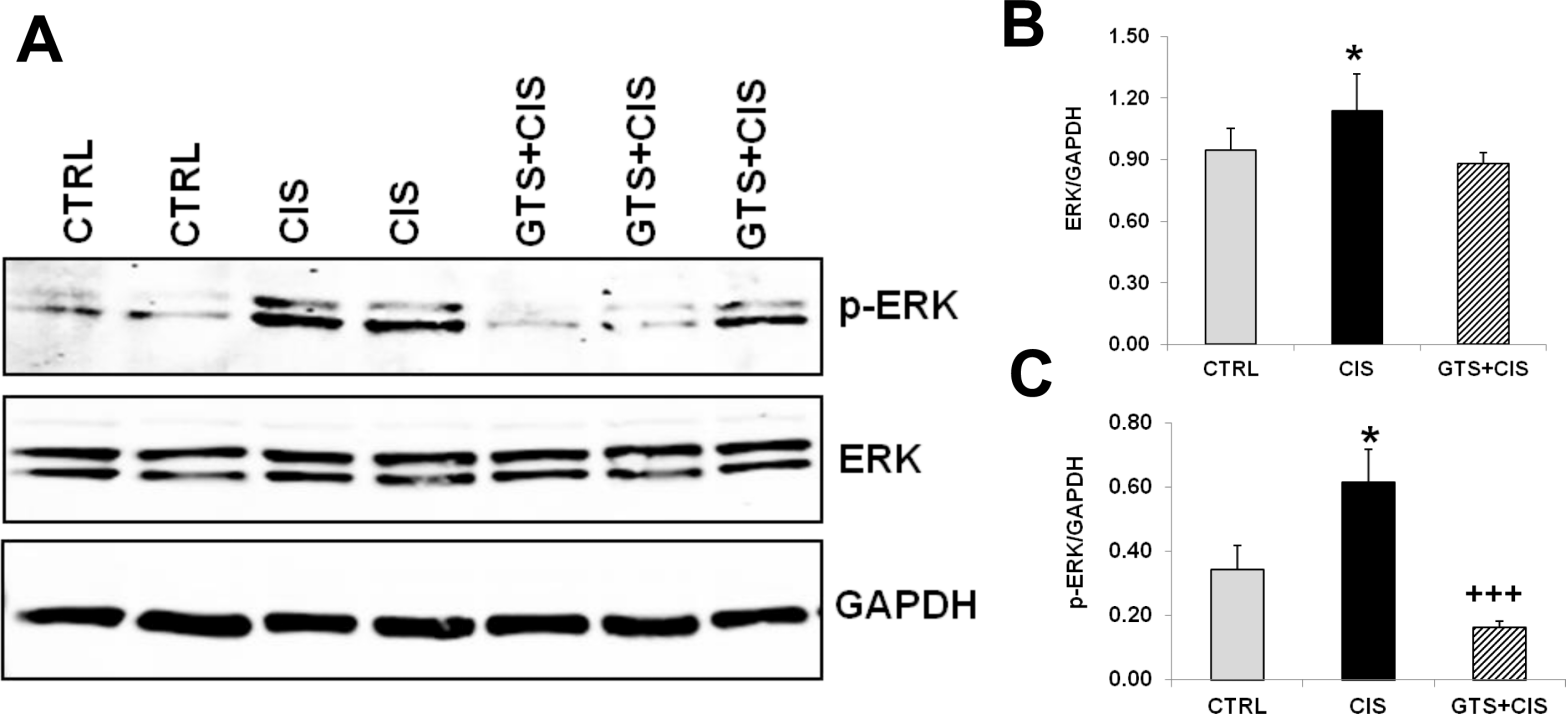
GTS-21 administration blocks cisplatin-induced renal ERK activation. Mice (CTRL, CIS and GTS+CIS groups, n = 8 per group) were treated and euthanized as described in the methods section and in Fig 1. ERK1/2 (ERK) protein expression and its phosphorylation (p-ERK) in the renal cortical tissues were measured by western blotting. GAPDH was used as loading control for normalization. Representative blots are shown in (A) and quantitation of the band densities are shown in (B, ERK/GAPDH) and (C, p-ERK/GAPDH). All data are expressed as mean band density (±SD). * = p<0.05 vs. CTRL, ^+++^ = p<0.001 vs. CIS.

Cisplatin regulates pro-inflammatory signaling in the kidneys, in part, via activation of extracellular signal-regulated kinase1/2 (p-ERK1/2)[27]. In our model, p-ERK1/2 and total ERK expression in the kidneys was enhanced by cisplatin administration; therefore we corrected p-ERK and total ERK for GAPDH levels (which was unaffected by cisplatin) (Fig 4A, 4B and 4C); GTS-21 treatment significantly blocked cisplatin-induced ERK phosphorylation (Fig 4A and 4C).

### GTS-21 alleviates cisplatin-induced renal ATP depletion and cellular oxidative stress

**Fig 5:**
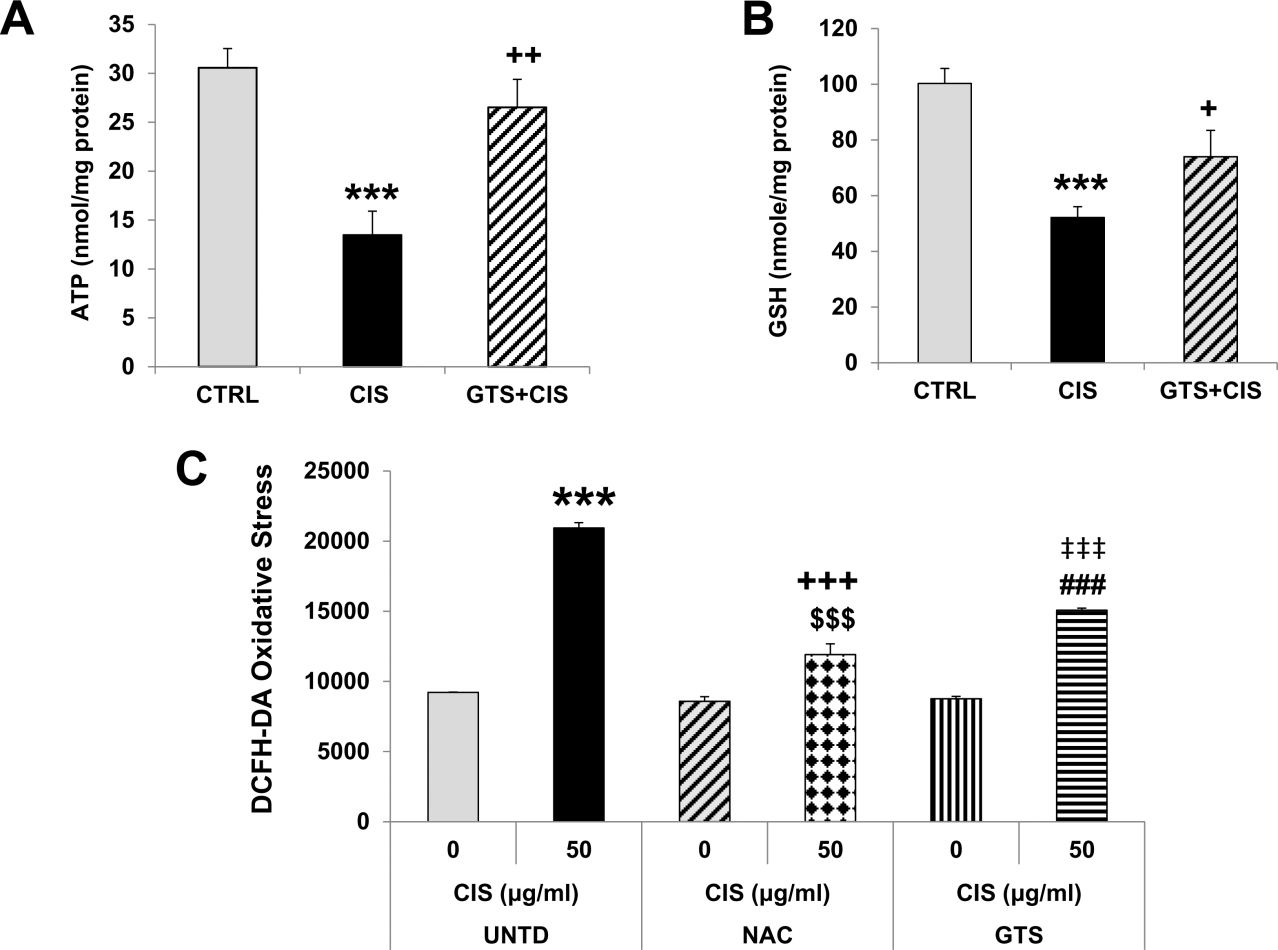
Cisplatin-induced renal ATP depletion and oxidative stress are improved by GTS-21. (A) ATP levels: Mice (CTRL, CIS and GTS+CIS groups, n = 8 per group) were treated and euthanized as described in the methods section and in Fig 1. Kidney homogenates were analyzed for renal ATP levels (shown as mean ATP concentration (nmol/mg, mean±SD), corrected for the protein levels. *** = p<0.001 vs. CTRL, ^++^ = p<0.01 vs. CIS. (B) GSH levels (reduced glutathione): LLC-PK_1_ cells were treated with GTS-21 (GTS, 1μM) for 1hr followed by cisplatin (CIS, 30μM) treatment or vehicle treatment (CTRL). After 20hrs, cells were harvested and the amount of GSH were assayed in the cell lysates to measure oxidative stress and corrected for the protein amount (nmol/mg, mean ±SD). *** = p<0.001 vs. CTRL, ^+^ = p<0.05 vs. CIS. (C) DCFH-DA assay: LLC-PK_1_ cells were treated with GTS-21 (GTS, 1μM) or NAC (2mM) or vehicle (UNTD). After 16hrs, cells were harvested and treated with either cisplatin (CIS, 50μg/mL) or vehicle (0μg/mL). After 3.5hrs, cells were labeled with DCFH-DA (20μM) for 20 min. Labeled cells were analyzed for oxidative stress by the conversion of the non-fluorescent DCFH-DA into a fluorescent compound DCF measured 30min later. Data (from 8–12 wells per condition) are shown as mean oxidative stress (or fluorescence) (±SD). *** = p<0.001 vs. vehicle, untreated (UNTD), ^+++^ = p<0.001 vs. vehicle NAC, ^‡‡‡^ = p<0.001 vs. vehicle GTS, ^$$$^ = p<0.001 vs. CIS (UNTD), ^###^ = p<0.001 vs. CIS (UNTD).

Energy depletion in kidney cells promotes injury and necrosis [28, 29]. We found that cisplatin significantly reduced kidney ATP concentrations by 60% vs. control kidneys (Fig 5A) and GTS-21 treatment significantly protected against renal ATP loss following cisplatin (Fig 5A).

To examine the effects of GTS-21 on renal oxidative stress we used the LLC-PK_1_ renal epithelial cell line. Cisplatin administration significantly reduced glutathione (GSH) levels in LLC-PK_1_ renal epithelial cells in vitro (Fig 5B), which was restored by the addition of GTS-21 prior to cisplatin (Fig 5B). Next, we examined the effect of GTS-21 on cisplatin-induced oxidative stress in real time using DCFH-DA, a fluorogenic dye that measures cellular levels of hydroxyl, peroxyl and other ROS [25, 26]. In the absence of cisplatin, basal oxidative stress in untreated renal epithelial cells was comparable to that observed following the addition of the potent anti-oxidant, NAC, or GTS-21 alone (Fig 5C). Cisplatin treatment of LLC-PK_1_ cells sharply increased oxidative stress by ~250% (Fig 5C). By contrast, both NAC and GTS-21 significantly blocked cisplatin-induced oxidative stress in renal epithelial cells (Fig 5C).

### GTS-21 blunts cisplatin-induced renal cell apoptosis

**Fig 6:**
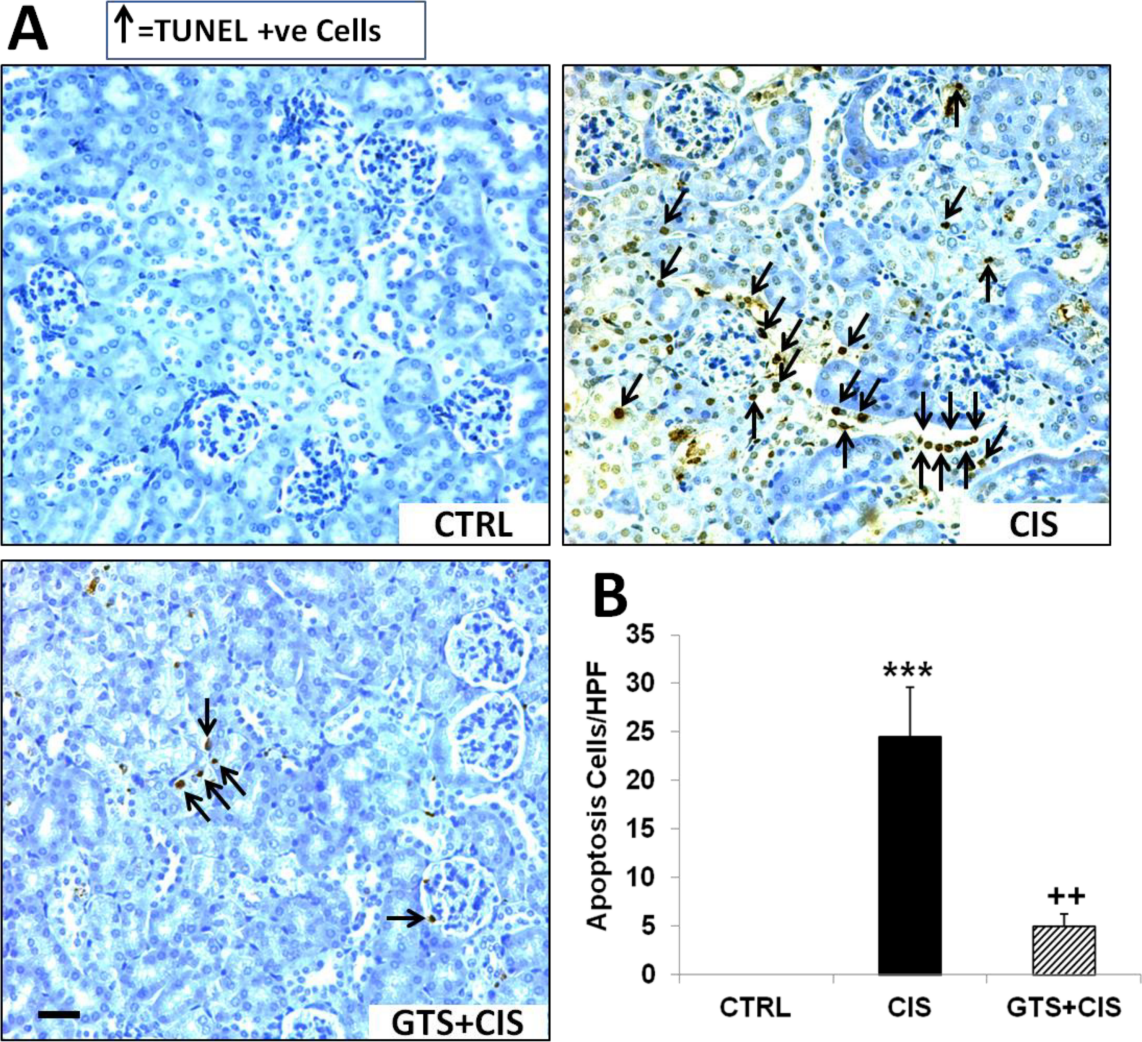
GTS-21 attenuates cisplatin-induced renal cell apoptosis. Mice (CTRL, CIS and GTS+CIS groups, n = 8 per group) were treated and euthanized as described in the methods section and in Fig 1. Renal cell apoptosis was measured by TUNEL staining and representative photomicrographs are shown (at 400x magnification; scale bar, 50 μm) in (A). Apoptosis was determined by counting the number of TUNEL positive cells per high power field (HPF) using random sections and the mean apoptosis scores (±SEM) are shown (B). *** = p<0.001 vs. CTRL, ^++^ = p<0.01 vs. CIS.

Next TUNEL staining was performed to assess apoptosis in the kidneys during cisplatin-AKI. As expected, cisplatin treatment induced significant renal cell apoptosis over control kidneys (Fig 6A and 6B). GTS-21 treatment prior to and post cisplatin administration significantly reduced the number of apoptotic cells in the kidneys when compared to kidneys obtained from mice treated with cisplatin alone (Fig 6A and 6B).

### Downregulation of renal cisplatin influx transporter expression by GTS-21

**Fig 7:**
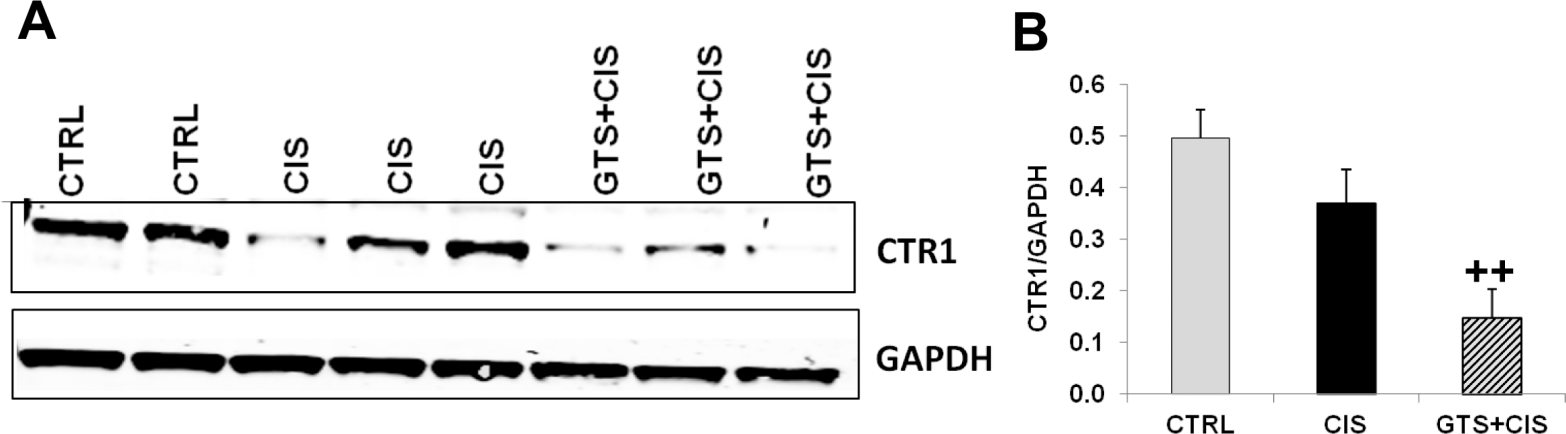
Downregulation of renal cisplatin influx transporter CTR1 expression by GTS-21. Mice (CTRL, CIS and GTS+CIS groups, n = 8 per group) were treated and euthanized as described in the methods section and Fig 1. Representative western blots showing renal CTR1 and GAPDH protein expression is shown in (A). GAPDH was used as loading control for normalization. Quantitation of band ratios: (B) CTR1/GAPDH (mean band density (±SD)) is shown. ^++^ = p<0.01 vs. CIS.

Pabla and colleagues demonstrated the role of CTR1 in mediating cisplatin uptake by renal tubular cells during nephrotoxicity in mice [30]. While cisplatin alone did not affect renal CTR1 protein expression in our model, GTS-21 treatment significantly reduced renal CTR1 protein levels in mice following cisplatin administration (Fig 7A and 7B).

### GTS-21 upregulates the expression of renal cisplatin efflux transporters

**Fig 8:**
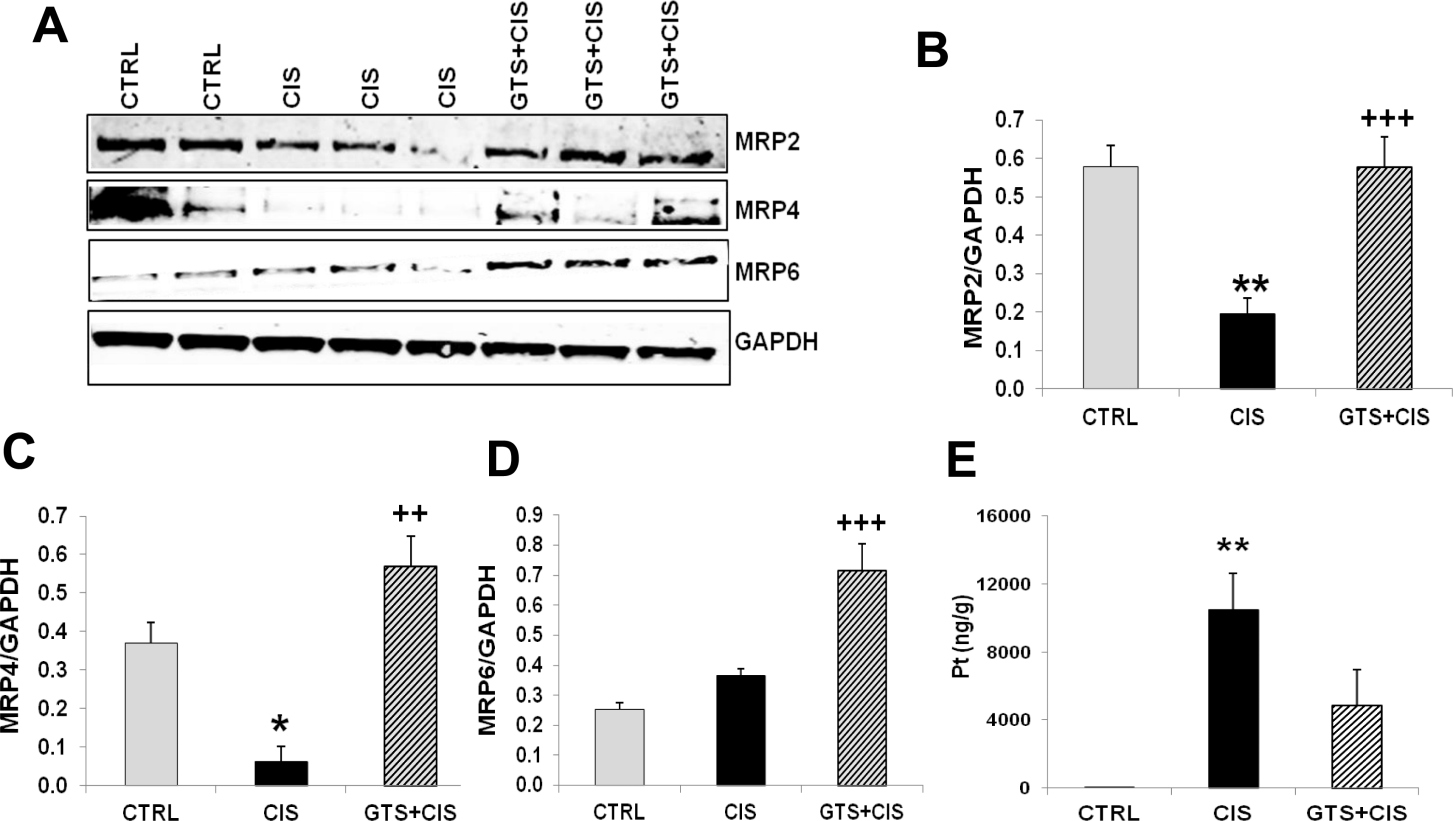
GTS-21 upregulates the renal expression of cisplatin efflux transporters. Mice (CTRL, CIS and GTS+CIS groups, n = 8 per group) were treated and euthanized as described in the methods section and Fig 1. Representative western blots for renal MRP2 (ABCC2), MRP4 (ABCC4) and MRP6 (ABCC6) protein expression are shown in (A). ABCC and MRP stand for ATP-binding cassette subfamily C and multidrug resistance-associated protein respectively. GAPDH was used as loading control for normalization. Quantitation of band ratios: (B) MRP2/GAPDH, (C) MRP4/GAPDH and (D) MRP6/GAPDH (mean band density (±SD) are shown. (E) Renal platinum (^195^Pt) accumulation, measured by ICP-MS, is shown as mean±SD (ng/g kidney tissue). ** = p<0.01 vs. CTRL, * = p<0.05 vs. CTRL, ^+++^ = p<0.001 vs. CIS, ^++^ = p<0.01 vs. CIS.

Cisplatin accumulation in the kidneys is the result of both cellular cisplatin uptake and cisplatin efflux via specific transporters, which are known as multi-drug resistance-associated proteins or MRPs. Administration of cisplatin alone significantly decreased renal MRP2 (ABCC2) and MRP4 (ABCC4) protein levels, while MRP6 (ABCC6) protein expression remained unchanged when compared to control mice (Fig 8A–8D). GTS-21 treatment prior to and post-cisplatin administration significantly increased MRP2, MRP4 and MRP6 protein levels in the kidneys when compared to cisplatin alone (Fig 8A–8D). Consistent with these observations, renal platinum (Pt) accumulation was evident following cisplatin treatment (Fig 8E). While Pt accumulation in the kidneys was reduced by GTS-21 treatment, this reduction was not statistically significant when compared to cisplatin alone (Fig 8E).

### GTS-21 does not inhibit cisplatin-mediated tumor cell killing *in vitro*

**Fig 9:**
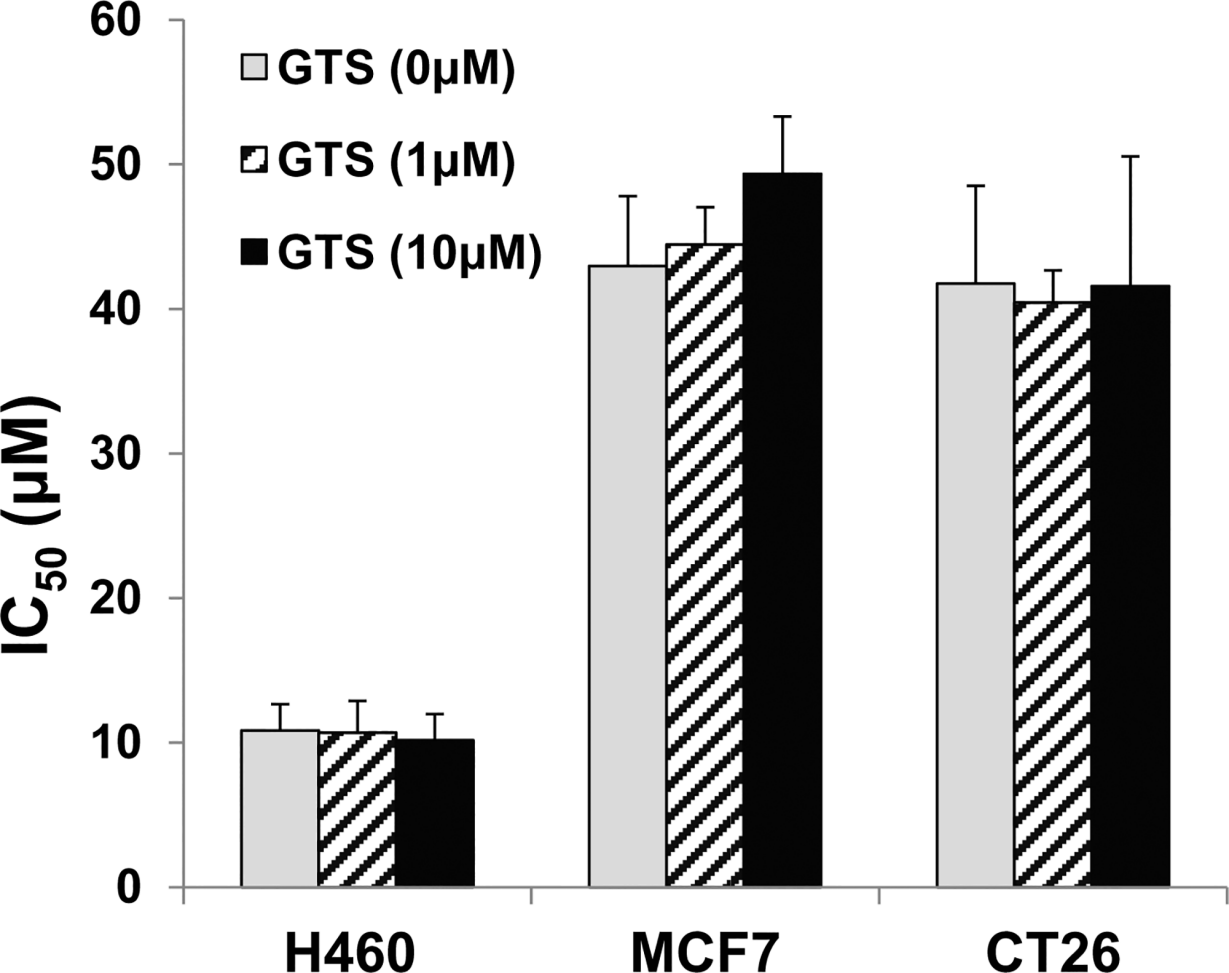
GTS-21 does not compromise cisplatin-induced tumor cell killing *in vitro*. Cancer cells (H460-lung; MCF7-breast, and CT26-colon) were treated with GTS-21 (at concentrations indicated) for 1hr followed by vehicle or cisplatin (0–230μM final) addition, and were assayed for cytotoxicity using the MTT assay 24 hrs later (n = 8–12 wells per condition). The IC_50_ values (mean±SD) for the tumor cell killing by cisplatin were calculated as described in the methods section.

Next, we used the H460 human lung cancer, MCF7 human breast cancer and CT26 mouse colon carcinoma cell lines to investigate whether GTS-21 treatment impaired the anti-tumor efficacy of cisplatin. As previously reported, all tumor cell lines were sensitive to cisplatin-mediated killing, with different sensitivities ranging from 10 to 43μM to kill one half of the cells (Fig 9). Pre-treatment of these tumor cell lines with GTS-21 (1μM and 10μM) prior to cisplatin addition did not significantly affect the concentrations of cisplatin required to kill one half of the tumor cells (IC_50_).

## Discussion

Although cisplatin is a highly effective chemotherapeutic agent, a dose of 50–100 mg/m^2^ causes approximately one-third of cancer patients to develop nephrotoxicity that requires dose reduction or drug discontinuation [5]. Currently, Amifostine is the only FDA-approved drug available for protecting against cisplatin-nephrotoxicity. However, its use is strictly limited by its high cost and serious side-effects [31], as well as its potential to diminish the anti-tumor potency of cisplatin [32, 33]. In the kidneys cisplatin causes DNA damage and mitochondrial dysfunction leading to the formation of ROS, which induce oxidative damage, inflammatory cytokine/chemokine production, tubule interstitial inflammation, and apoptosis/necrosis of renal tubular cells that together lead to AKI [4, 6]. Thus, inflammation and oxidative stress, two critical mediators of cisplatin-induced kidney injury, represent potential targets for renoprotective therapies.

Rodents and humans develop renal injury at fairly comparable cisplatin doses (15–20mg/kg in mice is equivalent to 45–60mg/mm^2^in humans) [34] and exhibit similar features of cisplatin-AKI, justifying the use of the mouse model. In our model, prophylactic GTS-21 significantly attenuated cisplatin-induced kidney injury, as determined by BUN and serum creatinine concentrations, as well as kidney injury scores determined by renal histopathology (Fig 1). These findings are consistent with the renoprotective effect of cholinergic agonists, nicotine and GTS-21, in experimental models of renal ischemia reperfusion injury via α7nAChR [13, 16] and septic AKI in mice [18]. More recent studies confirm that the activation of cholinergic anti-inflammatory pathway via vagus nerve stimulation and α7nAChR activation by agonists protects the kidneys during ischemia reperfusion injury [17, 35]. Furthermore, the cholinergic anti-inflammatory pathway induced by α7nAChR agonists or by vagus nerve stimulation abrogates oxidative stress and apoptosis in inflammatory models [13–15]. Cholinergic stimulation by electrical stimulation of the vagus nerve, in particular, has been shown to require an intact vagus nerve and spleen [9, 10]. However, vagus nerve cholinergic signaling alleviates the inflammatory state in hemorrhagic shock, ileus and other conditions via direct innervations of the liver, gastrointestinal tract and other organs, and the spleen is not required [36]. Our previous results using the rat kidney ischemia reperfusion injury model revealed that an intact vagus nerve was not required for renoprotection by GTS-21 [16] and support a localized renal effect. Future studies are needed to investigate the role of the spleen in mediating GTS-21’s effect in this model; these studies will be challenging because of the role of T lymphocytes in mediating cisplatin-induced kidney injury [37] and because splenectomy will dramatically affect immune status and alter circulating levels of B and T lymphocytes [38]. Although it is not known whether or not the spleen is required for GTS-21’s therapeutic effect, the vast majority of cancer patients receiving cisplatin have intact spleens.

Renal injury following cisplatin treatment is characterized by increased expression of inflammatory markers in the kidneys along with neutrophil infiltration [4, 6, 7, 39]. In our model, cisplatin-mediated renal damage was accompanied by local inflammation characterized by IL-1β, IL-6, and CXCL-1 production in the kidneys and renal *Ptgs2* (COX-2) mRNA expression (Fig 2). By contrast, we observed no effect of cisplatin on renal IL-10 levels and we were unable to detect TNFα in any kidney samples. IL-1β and IL-6 have been shown to be important inflammatory markers upregulated in the kidneys following cisplatin [40]. Likewise, CXCL1, produced during cisplatin-AKI potentially by T lymphocytes in the kidneys [41], is a well-known neutrophil chemoattractant capable of inducing apoptosis [42]. Previous studies report that α7nAChR^-/-^ mice show excessive CXCL1 production and uncontrolled neutrophil infiltration compared to wild-type mice after challenge with immune complexes [43]. In our model, GTS-21 significantly blocked cisplatin-induced IL-6 (by 80%), IL-1β (by 35%) and CXCL1 (by 70%). Interestingly, inhibition of IL-1β using IL-1 receptor antagonist alone and deletion of IL-6 alone (in IL-6^-/-^ mice) were not protective in a similar mouse model of cisplatin-AKI [40], suggesting that targeting multiple pathways is required for protection. Such a strategy is afforded by cholinergic stimulation via α7 nicotinic acetylcholine receptors. In addition to reducing IL-1β and IL-6, GTS-21 treatment significantly reduced cisplatin-mediated *Ptgs2* mRNA expression in the kidneys (Fig 2A). *Ptgs2* encodes the inducible form of cyclooxygenase-2 or COX-2, a target of numerous anti-inflammatory drugs because it is the main enzyme required for the conversion of arachidonic acid into prostaglandins. Renal COX-2 has been implicated in mediating cisplatin-AKI by promoting inflammation [44–46], while COX-2 inhibition reduces localized inflammatory infiltrates and protects against cisplatin-AKI [46–48]. As expected, renal neutrophil infiltration and enhanced renal MPO levels were observed during cisplatin-AKI (Fig 3). Treatment with GTS-21 significantly reduced both neutrophil accumulation and MPO concentrations in the kidneys of cisplatin-treated mice (Fig 3). A previous study by our group demonstrated that GTS-21 suppresses neutrophil accumulation in the kidneys following ischemia reperfusion injury [16], whereas the absence of the α7nAChR leads to exaggerated inflammatory infiltrate in the kidneys during anti-glomerular basement membrane (GBM) glomerulonephritis [49].

To the best of our knowledge, this is the first report describing the attenuation of cisplatin-induced AKI by activation of the cholinergic anti-inflammatory pathway. Ligation of α7nAChR downregulates pro-inflammatory cytokine expression through several molecular pathways, including NFκB [50], STAT3[51], and ERK1/2 [52–54]. Cisplatin activates cytokine production via ERK1/2 signaling in the kidneys [7, 27]. ERK1/2 is positioned upstream of the inflammatory pathway in cisplatin-AKI, and treatment with a MEK (MAPK/ERK kinase) inhibitor, UO126, suppresses cisplatin-induced renal inflammation and kidney cell apoptosis in mice [55, 56]. We found that cisplatin-induced both total ERK1/2 expression and phospho-ERK1/2 levels in the kidneys and that GTS-21 significantly blocked cisplatin-induced ERK1/2 activation without altering total ERK1/2 expression (Fig 4), suggesting that ERK1/2 may be an upstream target in this model. In addition to signaling inflammatory responses, ERK1/2 activation is associated with oxidant-mediated injury in renal epithelial cells following exposure to cisplatin [57].

Depletion of glutathione and other antioxidants by cisplatin leads to ROS accumulation and increased cellular oxidative stress associated with cisplatin-AKI [7]. Inside the cellular milieu, the aquated, positively charged electrophilic cisplatin accumulates in mitochondria and reduces the activity of the respiratory chain complexes (I–V) leading to excessive ROS formation, impaired ATP generation, and ultimately cell death [7]. Renal proximal tubular cells are one of the most metabolically active cell types which depend on mitochondrial ATP production and therefore, are most vulnerable to injury following mitochondrial damage [58, 59]. In this study GTS-21 prevented proximal tubular damage by attenuating cisplatin-mediated ATP depletion (Fig 5A), which was associated with reduced renal cell apoptosis (Fig 6A and 6B). Furthermore, GTS-21 suppressed cisplatin-mediated glutathione depletion (Fig 5B) and ROS formation (Fig 5C) in renal epithelial cells. Revealing the antioxidant property of GTS-21 is a novel finding, which corroborates the antioxidant property of other α7nAChR agonists [60–62]. These observations support improved mitochondrial function with GTS-21 treatment in this model but require additional studies to shed more light into the downstream effectors of GTS-21. Interestingly, mitochondrial α7nAChR expression has been reported [63–65]. Together with our previous studies showing the constitutive expression of α7nAChRs in rodent kidneys and renal tubular epithelial cells [22], as well as various cells in the kidney, including renal glomerular endothelial cells [18], the role of mitochondrially expressed α7nAChRs in mitigating cisplatin-mediated kidney injury should be investigated using pharmacological activators or vagus nerve stimulation.

The kidneys are a major target of cisplatin-induced toxicity because tubular epithelial cells express cisplatin transporters. The copper transporter, CTR1, is the main cisplatin influx transporter and deletion of CTR1 renders tumor cells more resistant to cisplatin in vitro and in vivo [66]. By contrast, overexpression of CTR1 promotes cisplatin uptake by cells, including renal tubular cells [30]. Renal accumulation of cisplatin following influx is balanced by efflux transporters, which pump cisplatin out of cells. The C subfamily of ATP-binding cassette transporters (ABCCs) is composed of more than a dozen multidrug resistance proteins or MRPs [67]. Interestingly, we observed that GTS-21 significantly reduced renal expression of the cisplatin uptake transporter, CTR1 (Fig 7) and significantly enhanced the expression of cisplatin efflux transporters MRP2, MRP4 and MRP6 by the kidneys (Fig 8A–8D). MRP2, 4, and 6 are expressed by renal proximal tubules and transport cisplatin [67]. MRP2 and MRP4 transporters efflux cisplatin-glutathione conjugates [67] and therefore their ability to efflux cisplatin may be further reduced by renal glutathione depletion observed during cisplatin-AKI. Consistent with the effects of GTS-21 on renal influx and efflux transporter expression, GTS-21 administration reduced, although not significantly, platinum (Pt) accumulation in the kidneys (Fig 8E). In our studies we may have missed the optimal time-point for assessing Pt because we euthanized mice 72hrs post-cisplatin administration and in rodents Pt levels peak in the kidneys approximately 2hrs post cisplatin administration and after that the levels fall and stabilize [68, 69].

Although GTS-21 is currently in clinical trials for numerous indications, including schizophrenia and Alzheimer’s disease, and appears to be relatively safe, the precise molecular mechanisms involved in its therapeutic effects are not completely understood. In this study, we link the therapeutic effect of GTS-21 to its anti-inflammatory and anti-oxidant activities, along with altered cisplatin transporter expression. However, it is not clear whether altered cisplatin uptake is a cause or consequence of GTS-21’s anti-inflammatory effect or whether GTS-21 directly or indirectly regulates cisplatin transporter expression. Our previous studies support that GTS-21 alters ubiquitin-proteasome activity (which may regulate cisplatin transporter levels) [18] and others have shown that anti-oxidant activity can regulate cisplatin transporter expression [70]. We propose that GTS-21 guards against cisplatin-mediated AKI by targeting multiple, protective pathways in the kidneys.

Here we demonstrate for the first time that treatment with GTS-21, a selective α7nAChR agonist, attenuates cisplatin-induced acute tubular injury and renal dysfunction in mice. Several studies have demonstrated that α7nAChRs mediate the effects of cholinergic agonists (e.g. GTS-21) [71, 72], including in the kidneys [13]. We did not assess the effect of cisplatin on α7nAChR expression in the kidneys. However, we administered GTS-21 prior to cisplatin. We hypothesize that prophylactic GTS-21 promotes a protective anti-inflammatory and anti-oxidant milieu in the kidneys that guards against cisplatin-AKI and reduces cisplatin accumulation in the kidneys through effects on cisplatin transporters and thus, support prophylactic GTS-21 administration in this model.

For clinical utility, protection of the kidneys should not compromise the effectiveness of cisplatin as an anti-tumor agent. Therefore, we examined whether GTS-21 compromised tumor killing by cisplatin using the H460 human lung cancer, MCF7 human breast cancer and CT26 mouse colon carcinoma cell lines. As previously reported, all tumor cell lines were sensitive to killing by cisplatin with IC_50_ values between approximately 10 and 40μM; the H460 cell line was the most sensitive (Fig 9). GTS-21 pre-treatment did not significantly affect the IC_50_ for cisplatin-mediated cancer cell killing (Fig 9). Although our preliminary studies do not suggest that GTS-21 protects tumor cells from cisplatin-mediated cytotoxicity, further pre-clinical studies are warranted to test the effect of GTS-21 on cisplatin-induced AKI in tumor-bearing mice to confirm our observations.

**Fig 10:**
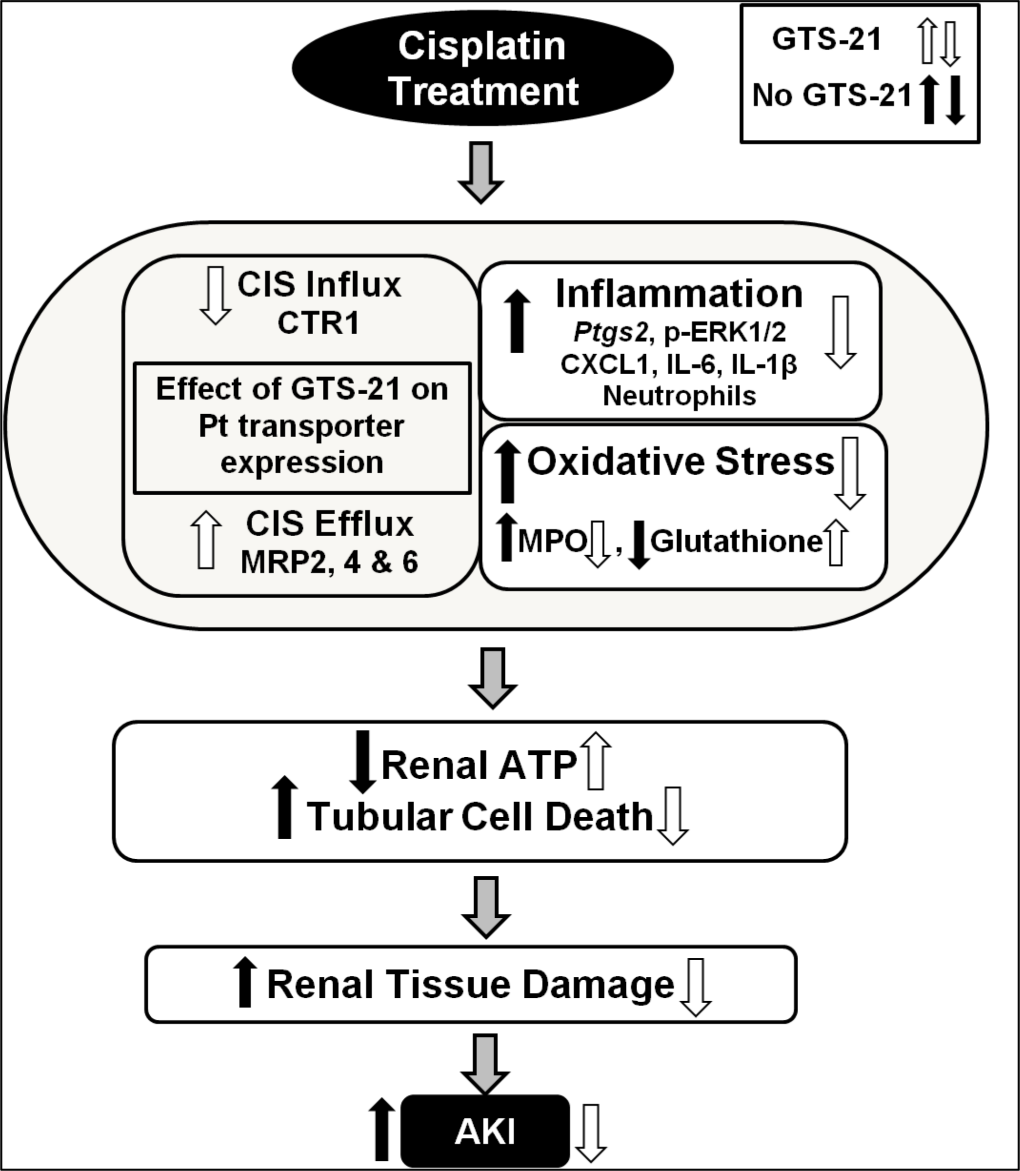
Proposed mechanisms by which GTS-21 attenuates cisplatin-induced AKI. Cisplatin treatment promotes renal inflammation and oxidative stress, renal cell apoptosis/necrosis, leading renal tissue damage and ultimately, AKI. GTS-21 treatment, acting through the cholinergic anti-inflammatory pathway, protects against cisplatin-induced AKI by reducing influx transporter expression while increasing CIS efflux transporter expression, and by modulating renal inflammation and oxidative stress pathways that lead to kidney cell death and AKI.

Based on our findings, we propose that GTS-21 exerts pleiotropic effects to protect against cisplatin-induced AKI (Fig 10). GTS-21 is renoprotective by decreasing cisplatin influx transporter expression and increasing cisplatin efflux transporter expression in the kidneys, which in turn reduces cisplatin accumulation. Prophylactic GTS-21 treatment reduces cisplatin-induced renal inflammation, improves oxidative stress and inhibits ATP depletion leading to attenuated renal cell apoptosis, renal tissue damage and eventually attenuation of AKI. Prior studies demonstrate that GTS-21 reduces inflammatory mediator production [18, 21, 73], which support the effect of GTS-21 on cisplatin-induced inflammatory mediator production in our model. Additional reports indicate that GTS-21 reduces inflammation by targeting upstream mediators (e.g. ERK1/2)[74]. Consistent with this study, we show that GTS-21 significantly reduced cisplatin-induced renal ERK1/2 activation (Fig 4), which has been implicated in mediating cisplatin-induced renal inflammation and renal cell apoptosis [55, 56]. Furthermore, we show that GTS-21 decreased cisplatin-mediated glutathione depletion and cisplatin-mediated ROS production by renal epithelial cells, supporting its anti-oxidant activities. Thus, GTS-21 appears to exerts its renoprotective effects via regulating multiple pathways that converge to produce kidney injury following cisplatin exposure, including anti-inflammatory and anti-oxidant pathways, and by modulating cisplatin accumulation in the kidneys via regulating influx/efflux transporter expression. These findings along with prior studies revealing the renoprotective activity of GTS-21 and other cholinergic agonists strongly support additional pre-clinical studies exploring the therapeutic potential of α7nAChR agonists, as well as vagus nerve stimulation, which has shown some success for treating patients with rheumatoid arthritis [75] and Crohn’s disease [76], for protecting against cisplatin-mediated AKI.

## Conclusions

Currently, there are no therapies available to treat or prevent cisplatin-induced AKI. Our results show that cisplatin-mediated AKI can be significantly reduced by GTS-21 administration in mice. Additional *in vitro* studies with multiple tumor cell lines (lung, breast and colon) show that GTS-21 does not compromise cisplatin’s anti-tumor effects. These findings warrant additional studies to identify the precise mechanisms of GTS-21’s renoprotective effects and to explore the use of prophylactic GTS-21 treatment for suppressing AKI following cisplating administration.
